# Conicity-index predicts all-cause mortality in Chinese older people: a 10-year community follow-up

**DOI:** 10.1186/s12877-022-03664-6

**Published:** 2022-12-16

**Authors:** Anhang Zhang, Yingnan Li, Shouyuan Ma, Qiligeer Bao, Jin Sun, Shuang Cai, Man Li, Yongkang Su, Bokai Cheng, Jing Dong, Yan Zhang, Shuxia Wang, Ping Zhu

**Affiliations:** 1 Medical School of Chinese PLA & Chinese, PLA General Hospital, Beijing, 100853 China; 2 Department of Geriatrics, The Second Medical Center & National Clinical Research Center for Geriatric Diseases, Chinese PLA General Hospital, 28 Fuxing Road, Beijing, 100853 China; 3 Department of Gastroenterology, the Second Medical Center, Chinese PLA General Hospital, Beijing, 100853 China; 4 Department of Geriatric Cardiology, the Second Medical Center, Chinese PLA General Hospital, Beijing, 100853 China; 5 Department of Outpatient, the First Medical Center, Chinese PLA General Hospital, Beijing, 100853 China

**Keywords:** Conicity-index (C-index), Abdominal obesity (AO), All-cause mortality, Community, Older people

## Abstract

**Background:**

Abdominal obesity (AO) has been regarded as the most dangerous type of obesity. The Conicity-index (C-index) had a high ability to discriminate underlying AO. The purpose of this study was to determine the ability of C-index to predict all-cause mortality among non-cancer Chinese older people.

**Methods:**

The participants were residents of the Wanshou Road community in Beijing, China. Receiver operating curve (ROC) curves were used to determine the sensitivity and specificity of the best cut-off values for different anthropometric measures for predicting all-cause mortality. The area under the curve (AUC) of the ROC curves were calculated to compare the relative ability of various anthropometric measures to correctly identify older people in the community where all-cause mortality occurs. Included subjects were grouped according to C-index tertiles. The association between C-index and all-cause mortality was verified using Kaplan–Meier survival analysis and different Cox regression models.

**Results:**

During a mean follow-up period of 9.87 years, 1821 subjects completed follow-up. The average age was 71.21 years, of which 59.4% were female. The ROC curve results showed that the AUC of the C-index in predicting all-cause mortality was 0.633. Kaplan–Meier survival curves showed a clear dose–response relationship between C-index and all-cause mortality. With the increase of C-index, the survival rate of the study population showed a significant downward trend (*P* < 0.05). Adjusted for age, gender, hip circumference, systolic blood pressure, diastolic blood pressure, fasting blood glucose (FBG), 2-h postprandial blood glucose (2hPG), glycosylated hemoglobin, high-density lipids protein (LDL), triglyceride, serum creatinine, serum uric acid, urine albumin-creatinine ratio (UACR), Mini-Mental State Examination (MMSE), smoking history, and drinking history, COX regression analysis showed that in the model adjusted for all covariates, the risk of all-cause mortality in tertile 3 was 1.505 times that in tertile 1, and the difference was statistically significant.

**Conclusions:**

The C-index is an independent risk factor for all-cause mortality in the non-cancer Chinese older people.

## Introduction

Obesity is a complex multifactorial disease. Nearly one-third of the world's population is now classified as overweight or obese [1, 2]. Of the more than two billion people worldwide who are overweight or obese, 62% of them live in developing countries [3]. In 2014, China became the country with the largest number of obese people in the world, with a total of 90 million obese individuals, accounting for 14% of the world's obese people [4]. In addition, abdominal obesity has been regarded as the most dangerous type of obesity, and studies have shown that the prevalence of abdominal obesity in China reached 31.5% from 2013 to 2014 [5, 6]. Abdominal obesity has been linked to a number of chronic diseases, such as type 2 diabetes mellitus (T2DM), hypertension, and atherosclerosis [7–9]. Studies have also shown that obesity is associated with all-cause mortality [10–13]. Quantification of visceral adiposity is best determined by imaging studies, such as computed tomography (CT), which is the gold standard method but requires high cost, difficult manipulation, and radiation exposure [14, 15]. On the other hand, anthropometric clinical indicators are readily available and, if accurate, can provide diagnostic possibilities in primary care and follow-up without any inconvenience. However, the most widely used measure of obesity, body mass index (BMI), appears to be rather insensitive to abdominal fat deposition. Because it does not reflect an individual's fat distribution or differentiate between fat mass and muscle mass, BMI has its limitations [16, 17]. Therefore, many special indexes sensitive to abdominal fat have been studied, including waist circumference (WC), abdominal volume index (AVI), body roundness index(BRI), lipid accumulation products (LAP), waist-height ratio (WHtR), and conicity-index (C-index), etc. [18–20]. The C-index is based on the assumption that individuals with more fat around the abdomen are biconical, while those with less fat around the midsection are cylindrical [17]. This index involves variables such as weight, height, and WC. C-index was determined as an indicator of body fat distribution, and their values increased with the accumulation of fat in the abdominal region [7]. Numerous studies have pointed to the high ability of C-index in distinguishing underlying abdominal obesity (AO) [14, 21]. Abdominal obesity can be highly discriminated by the anthropometric measure C-index, which is considered to be a major cardiovascular risk factor [22]. C-index has also been found to be associated with hypertriglyceridemia, decreased high-density lipoprotein, and elevated low-density lipoprotein, which may also increase the risk of all-cause mortality [23]. However, there is still a gap in research on whether the C-index can predict all-cause mortality in older community populations. The purpose of this study was to fill a research gap by determining the ability of the C-index to predict all-cause mortality in community-based older people through follow-up.

## Methods

### Subject

**Fig. 1:**
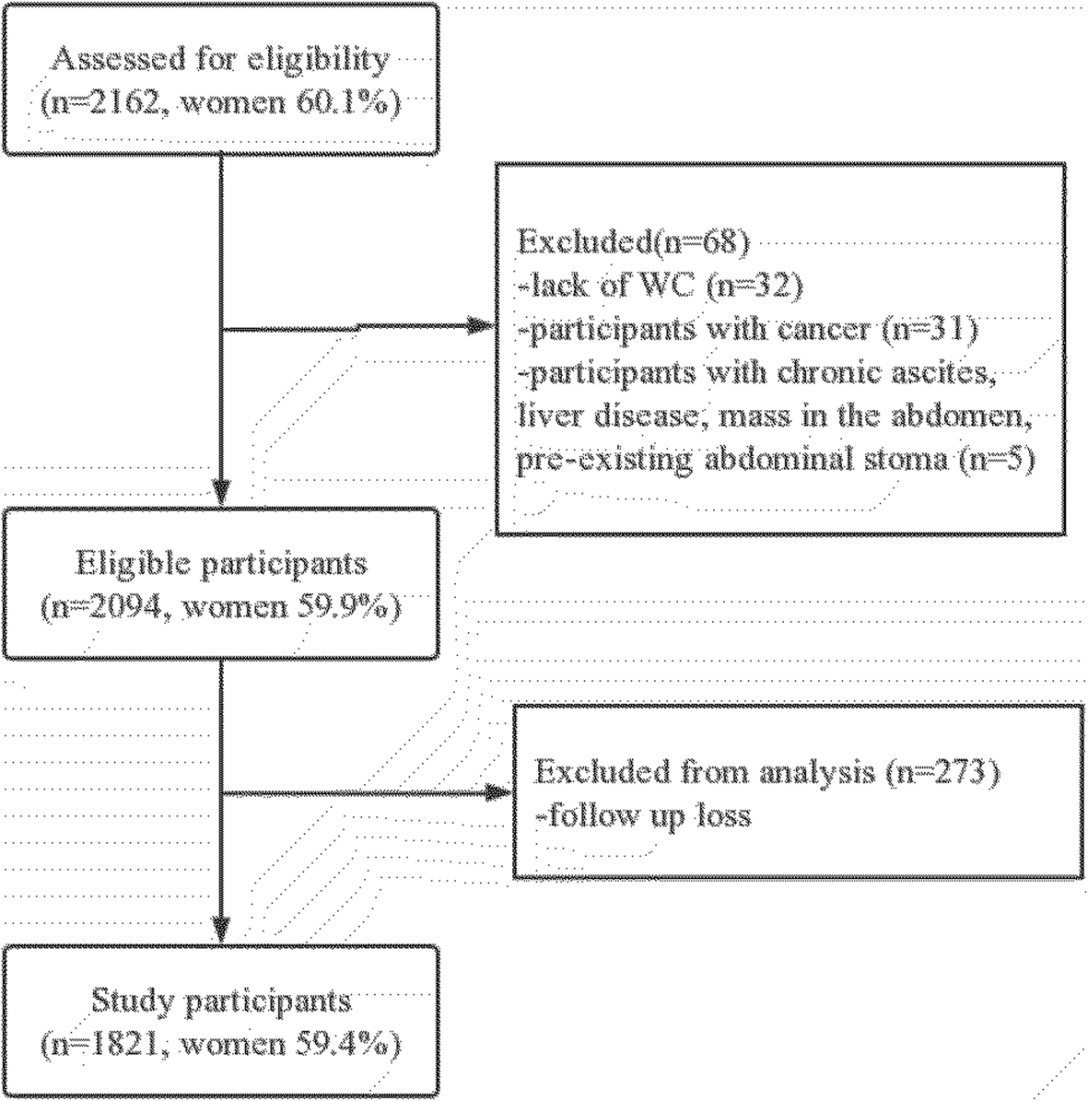
Recruitment process for the study population. WC: waist circumference

This is a cohort study of people over 60 years old on Wanshou Road, a representative urban residential area in Beijing. Sampling and research methods have been reported in previously published articles [24–26]. A population-based cross-sectional survey of the participants in the Wanshou Road community, a representative urban residential area in Beijing, was conducted using a two-stage hierarchical cluster sampling method. Between September 2009 and June 2010, a total of 2162 residents aged 60–95 years were selected and invited for screening. Figure 1 shows the recruitment process for the study population. The study protocol was reviewed and approved by the Ethics Committee of the Chinese People's Liberation Army General Hospital. All participants gave informed consent before being recruited. All investigators were trained at the PLA General Hospital (Beijing, China) and passed the test.

### Data collection

Men and women completed detailed baseline health and lifestyle questionnaires, and 2162 participated in a health check-up performed by trained nurses using standard procedures [24]. Baseline health data and lifestyle questionnaires were used to obtain some information about the participants, including alcohol and smoking status, history of diabetes, history of hypertension, history of coronary heart disease, National Institutes of Health Stroke Scale (NIHSS), Mini-Mental State Examination (MMSE), and activities of daily living (ADL), etc. [26]. Height is measured with an independent distance measuring device, accurate to 0.1 cm. Weight was calculated with an electronic scale, accurate to 0.1 kg. WC was measured horizontally between the lower edge of the ribs and the iliac crest using a piece of non-elastic tape and hip circumference (HC) was measured at the widest part of the hip and at the top of the iliac crest to an accuracy of 0.1 cm. The participant empties the bladder 30 min before the blood pressure measurement. Rest in a backed chair, allow the whole body to relax and measure the blood pressure with the sphygmomanometer in a quiet environment. The systolic and diastolic blood pressures were measured twice with a time interval of five minutes, and the average of the two was used for analysis. Hypertension is doctor-diagnosed hypertension or systolic blood pressure ≥ 140 mmHg or diastolic blood pressure ≥ 90 mmHg or received antihypertensive medication. Type 2 diabetes mellitus refers to T2DM diagnosed by a doctor or fasting blood glucose value ≥ 7.0 mmol/L after fasting for more than eight hours, or blood glucose ≥ 11.1 mmol/L after 2 h of oral glucose, or blood glucose ≥ 11.1 mmol/L at any time, or insulin at the time of admission. Fasting blood glucose, 2-h postprandial blood glucose, glycosylated hemoglobin, serum total cholesterol, high-density lipoprotein cholesterol, low-density lipoprotein cholesterol, and triglycerides were detected with an automatic biochemical analyzer. All on-site urine samples were taken, the urine albumin concentration (mg/l) and the urine creatinine concentration (g/l) were measured, and the urine albumin-creatinine ratio (mg/g) was calculated [26]. All biochemical analyses were performed in the Department of Biochemistry, Chinese People's Liberation Army General Hospital. 1821 of the subjects agreed to participate in telephone follow-up or signed written informed consent for community field follow-up, which constituted our study population. There were no differences between responders and non-responders in age and gender. This study was ethically approved by the Ethics Committee of the Chinese People's Liberation Army General Hospital.

### Anthropometric measurements for identifing obesity

BMI was calculated with the following formula:

$$\mathrm{BMI}=\mathrm{Weight}\left(\mathrm{Kg}\right)/{\mathrm{Height}}^{2}\left({\mathrm{m}}^{2}\right)$$

C-index was calculated with the following formula:

$$\mathrm C-\mathrm{index}=0.109^{-1}{\mathrm{WC}\left(\mathrm m\right)\lbrack\mathrm{Weight}\left(\mathrm{Kg}\right)/\mathrm{Height}\left(\mathrm m\right)\rbrack}^{-1/2}$$

BRI was calculated with the following formula:

$$\mathrm{BRI}=364.2-365.5{\left[1-{\uppi }^{-2}{\mathrm{WC}}^{2}\left(\mathrm{m}\right){\mathrm{Height}}^{-2}\left(\mathrm{m}\right)\right]}^{1/2}$$

WHtR was calculated with the following formula:

$$\mathrm{WHtR}=\mathrm{WC}\left(\mathrm{cm}\right)/\mathrm{Height}\left(\mathrm{cm}\right)$$

AVI was calculated with the following formula:

$$\mathrm{AVI}=\left[2\times {\mathrm{WC}}^{2}\left(\mathrm{cm}\right)+0.7\times {\left(\mathrm{WC}\left(\mathrm{cm}\right)-\mathrm{HC}\left(\mathrm{cm}\right)\right)}^{2}\right]/1000$$

LAP was calculated with the following formula:

$$\mathrm{Males}:\mathrm{ LAP}=\left(\mathrm{WC}\left(\mathrm{cm}\right)-65\right)\times \mathrm{TG}\left(\mathrm{mmol}/\mathrm{L}\right)$$

$$\mathrm{Females}:\mathrm{ LAP}=\left(\mathrm{WC}\left(\mathrm{cm}\right)-58\right)\times \mathrm{TG}\left(\mathrm{mmol}/\mathrm{L}\right)$$

### Grouping and endpoints

The eligible subjects were divided into three groups according to C-index tertiles. Endpoint deaths were defined as indicator events occurring between baseline and follow-up up to December 31, 2020. During follow-up, the primary concern was all-cause mortality. In these analyses, all-cause deaths were defined as deaths from any cause that resulted from phone calls or on-site follow-up provided by family members. For some team members who lost their contact numbers or changed their home addresses, our staff sought help from community workers and police officers in the Population management and Archives Office of Wanshou Road Police Station [26].

### Statistical analysis of data

All categorical data are presented as percentage (%), continuous data are presented as mean ± standard deviation. The Kolmogorov-Smirnoff test was used to test whether the data had a normal distribution. Baseline characteristics of the study population were analyzed using a one-way analysis of variance (ANOVA) test. For post hoc multiple comparisons, the Tamhane's T2 test is used to assume that the variances are not equal, while the LSD test is used to assume that the variances are equal. Receiver operating characteristic (ROC) curves were used to determine the sensitivity and specificity of optimal cutoff values for obesity measures (BMI, WC, AVR, BRI, LAP, WHtR, and C-index) for predicting all-cause mortality. The area under the curve (AUC) of the ROC curve was calculated to compare the relative ability of various anthropometric measures to correctly identify the occurrence of all-cause mortality in older people in the community.

The chi-square test was used to compare all-cause mortality after ten years in different groups. Cumulative probabilities were compared using the Kaplan–Meier method and the log-rank test. In the Cox proportional hazards model analysis, hazard ratios (HR) and 95% confidence intervals (CI) were calculated. Model 1: All covariates were not adjusted; Model 2: Adjusted for age and gender; Model 3: Adjusted for age, gender, hip circumference, systolic blood pressure, diastolic blood pressure, fasting blood glucose, 2-h postprandial blood glucose, glycosylated hemoglobin, high-density lipoprotein, triglyceride, serum creatinine, serum uric acid, urine albumin-creatinine ratio, MMSE, smoking history, and drinking history. A multivariate logistic regression model was established to show the relationship between C-index and all-cause mortality. In the Cox regression model, tolerance (Tol) and variance inflation factors (VIF) are calculated for each covariate to test for multicollinearity, ensuring that there is no prior multicollinearity problem for each covariate. The Omnibus test was used to test the model coefficients, and the results were analyzed by the survival analysis function. In all hypothesis tests, the risk of type 1 error was a priori set at *P* ≥ 0.05. All statistical tests were two-sided with a significance level of α = 0.05. Statistical analysis was performed using SPSS software (version 26.0).

## Results

### Baseline characteristics

Among the 1821 study populations, 59.4% were female, grouped by C-index tertiles (Tertile 1: C-index < 1.249; Tertile 2: 1.249 ≤ C-index < 1.312; Tertile 3: C-index ≥ 1.312), and a one-way analysis of variance (ANOVA) test was used to analyze the baseline characteristics of the study population. Table 1 shows the baseline characteristics of the different groups.

**Table 1:** Baseline characteristics of the study population (all men and women), grouped by C-index tertiles

**Parameter**	All patients (*n* = 1821)	Tertile 1 C-index < 1.249 (*n* = 620)	Tertile 2 1.249 ≤ C-index < 1.312 (*n* = 602)	Tertile 3 C-C-C-C-index ≥ 1.312 (*n* = 599)	*P* value
Age (years)	71.21 ± 6.80	69.22 ± 6.32	70.88 ± 6.69	73.57 ± 6.67	< 0.001
Sex (females %)	59.4	71.6	55.8	50.4	< 0.001
Height (cm)	160.55 ± 8.16	159.01 ± 7.35	161.37 ± 8.11	161.31 ± 8.78	< 0.001
Weight (kg)	64.53 ± 10.91	61.33 ± 9.76	65.89 ± 10.16	66.49 ± 12.00	< 0.001
BMI (kg/m^2^)	24.98 ± 3.43	24.22 ± 3.32	25.28 ± 3.33	25.46 ± 3.52	< 0.001
Waist (cm)	88.12 ± 9.30	80.61 ± 7.34	88.86 ± 6.16	95.14 ± 7.82	< 0.001
Hip (cm)	98.28 ± 8.05	94.70 ± 7.55	99.12 ± 6.91	101.13 ± 8.23	< 0.001
Systolic pressure (mmHg)	138.36 ± 19.25	136.47 ± 18.16	138.62 ± 19.48	140.07 ± 19.92	0.004
Diastolic pressure (mmHg)	77.15 ± 7.74	75.85 ± 9.64	77.85 ± 9.77	77.82 ± 9.66	< 0.001
FBG (mmol/L)	6.04 ± 1.54	5.84 ± 1.24	6.06 ± 1.61	6.22 ± 1.71	< 0.001
2hPG (mmol/L)	8.15 ± 3.26	7.71 ± 2.91	8.14 ± 3.32	8.66 ± 3.52	< 0.001
Glycosylated hemoglobin (%)	6.08 ± 1.22	5.95 ± 0.94	6.09 ± 1.28	6.21 ± 1.39	< 0.001
Total cholesterol (mmol/L)	5.24 ± 1.01	5.28 ± 1.00	5.30 ± 1.05	5.14 ± 0.97	0.011
HDL(mmol/L)	1.42 ± 0.38	1.50 ± 0.40	1.38 ± 0.35	1.36 ± 0.39	< 0.001
LDL(mmol/L)	3.23 ± 0.86	3.23 ± 0.86	3.31 ± 0.86	3.13 ± 0.84	0.001
Triglyceride (mmol/L)	1.66 ± 0.91	1.57 ± 0.91	1.68 ± 0.95	1.73 ± 0.85	0.006
Serum creatinine (umol/L)	74.30 ± 22.27	71.13 ± 20.61	73.63 ± 18.20	78.21 ± 26.70	< 0.001
Blood uric acid (umol/L)	308.93 ± 88.56	290.97 ± 77.87	308.40 ± 89.86	327.83 ± 93.14	< 0.001
UACR	36.01 ± 193.17	22.08 ± 87.15	36.54 ± 228.95	44.73 ± 190.95	0.017
NIHSS	0.12 ± 0.55	0.08 ± 0.41	0.09 ± 0.37	0.19 ± 0.77	0.007
MMSE	26.96 ± 3.36	27.45 ± 3.17	27.08 ± 3.24	26.33 ± 3.57	< 0.001
ADL	98.97 ± 5.32	99.30 ± 3.88	98.84 ± 6.29	98.79 ± 5.49	0.109
Smoking (%)	30.2	23.4	29.7	37.9	< 0.001
Drinking (%)	25.0	20.0	25.9	29.2	0.001
CHD (%)	23.6	21.9	23.1	25.9	0.252
Stroke (%)	12.7	10.2	12.5	15.2	0.029
HT (%)	55.0	52.3	55.0	57.8	0.155
T2DM (%)	18.5	14.8	18.1	22.5	0.002
eGFR	60.5 ± 36.28	59.7 ± 34.85	59.7 ± 26.51	62.1 ± 45.18	0.414

*FBG* Fasting blood glucose, *2hPG* 2-h post-meal blood glucose, *HDL* High-density lipoprotein, *LDL* Low-density lipoprotein, *UACR* Urinary albumin to creatinine ratio, *NIHSS* National Institutes of Health Stroke Scale, *MMSE* Mini-Mental State Examination (MMSE), *ADL* Activity of Daily Living, *CHD* Coronary Heart Disease, *HT* Hypertension, *T2DM* Type 2 diabetes mellitus, *eGFR* Estimated glomerular filtration rate

### Obesity index and all-cause death

**Fig. 2:**
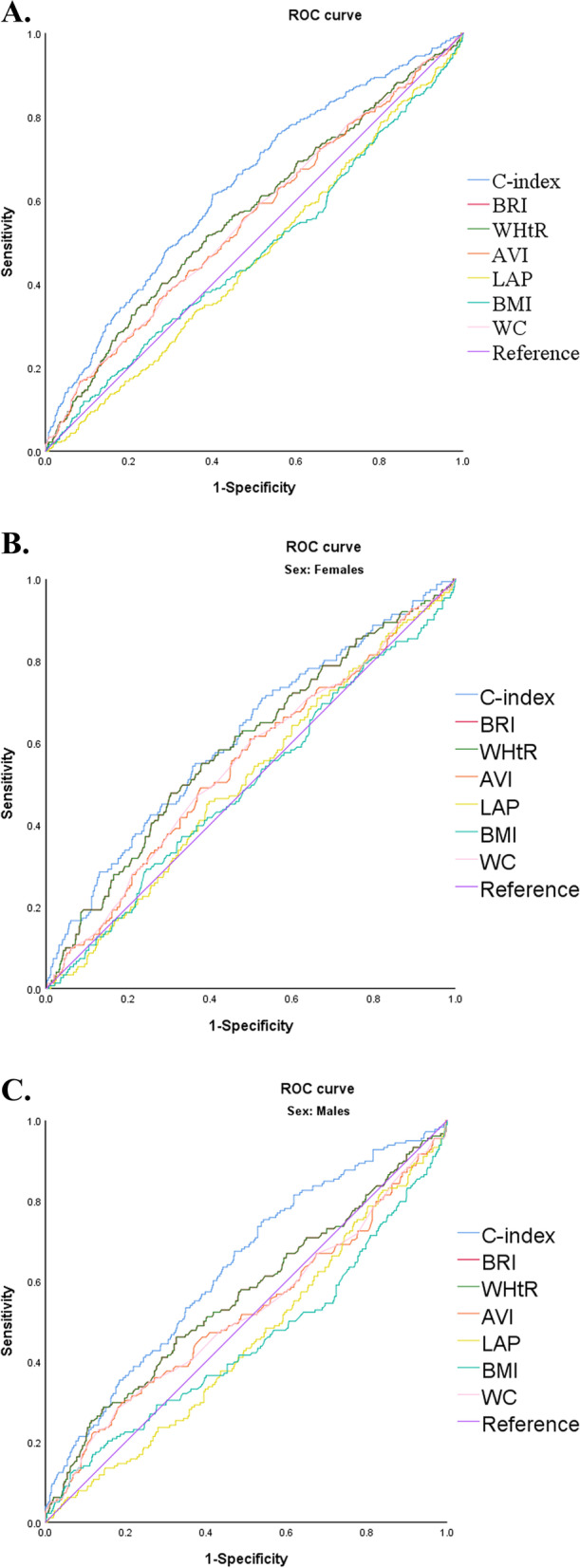
ROC curves of different obesity indices and all-cause mortality. **A** all subjects; **B** male subjects; **C** female subjects. C-index: conicity-index; BRI: body roundness index; WHtR: waist-height ratio; AVI: abdominal volume index; LAP: lipid accumulation products; WC: waist circumference

Figure 2 shows the ROC curves and their respective areas under the curve (AUC) for different obesity indices and all-cause mortality for all subjects, male subjects, and female subjects, respectively. Compared with various commonly used clinical obesity indices, C-index showed better ability in predicting all-cause mortality in older people in the community, with a larger area under the curve.

### C-index and all-cause death

During a mean follow-up period of 9.87 years (18,029.5 person-years of follow-up), 1821 subjects were followed, of whom 333 died (179 men and 154 women). Overall population mortality, and crude all-cause mortality for men and women were 182.9/1000, 242.2/1000, and 142.3/1000, respectively. Table 2 shows the comparison of all-cause mortality in different groups in the chi-square test.

**Table 2:** Comparison of all-cause mortality in different groups by chi-square test

Survive Crosstabulation								
	C-index						Total	
	Tertile 1		Tertile 2		Tertile 3			
	Count	% within group	Count	% within group	Count	% within group	Count	% within group
Survive	553	89.20%	496	82.40%	439	73.30%	1488	81.70%
All-cause deaths	67	10.80%	106	17.60%	160	26.70%	333	18.30%
Total	620	100.00%	602	100.00%	599	100.00%	1821	100.00%

**Fig. 3:**
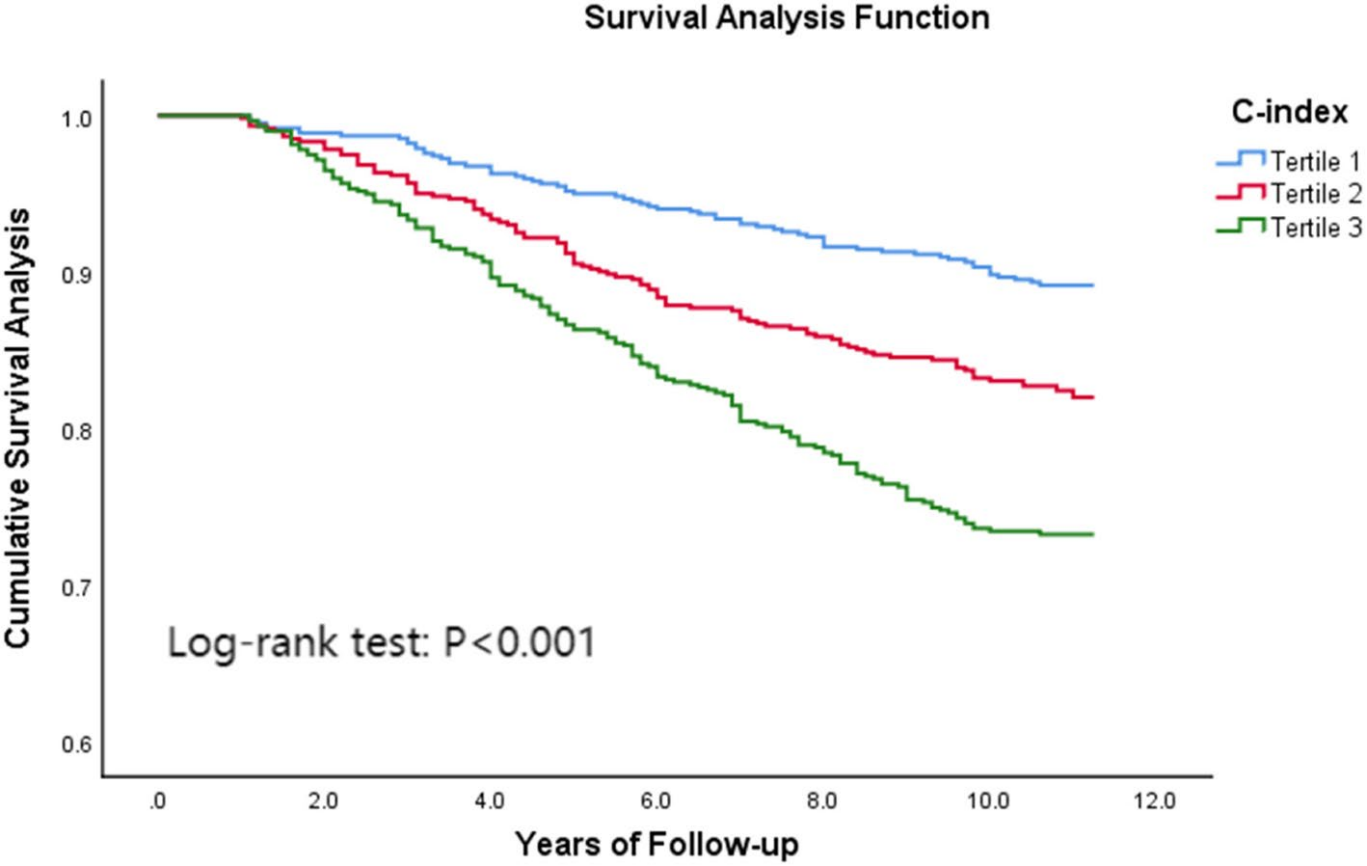
Kaplan–Meier survival curves under different C-index groups. Tertile 1: C-index < 1.249; Tertile 2: 1.249 ≤ C-index < 1.312; Tertile 3: C-index ≥ 1.312

Figure 3 shows the Kaplan–Meier survival curves under different C-index groups. There was a significant dose–response relationship between C-index and all-cause mortality. With the increase of C-index, the survival rate of the study population showed a significant downward trend, and the *P* values were all less than 0.001 on the Log-Rank test, Breslow test, and Tarone-Ware test.

To investigate the effect of C-index on all-cause mortality, we fit a Cox proportional hazards model to the data. We built three Cox proportional hazards models. As can be seen from Table 3, in Model 1 without adjusting for any covariates, the risk of all-cause mortality for Tertile 3 was 2.730 times that of Tertile 1, and the risk of all-cause death for Tertile 2 was 1.706 times that of Tertile 1. After adjusting for age and sex, in model 2, the proportion of the risk of all-cause mortality decreased in Tertile 3, which was 1.656 times that of Tertile 1. In Model 3, after adjusting for all relevant covariates that may affect survival, the risk of all-cause mortality in Tertile 3 was 1.505 times that of Tertile 1, and the difference was still statistically significant.

**Table 3:** Cox regression analysis of the relationship between C-index and all-cause mortality

		C-index			
		Tertile 1	Tertile 2	Tertile 3	P for trend
HR (95%CI)	Model 1	1 (Reference)	1.706* (1.256, 2.316)	2.730* (2.052, 2.631)	< 0.001
	Model 2	1 (Reference)	1.379* (1.012, 1.878)	1.656* (1.234, 2.223)	0.001
	Model 3	1 (Reference)	1.233 (0.863, 1.761)	1.505* (1.049, 2.157)	0.029

Tertile 1: C-index < 1.249; Tertile 2: 1.249 ≤ C-index < 1.312; Tertile 3: C-index ≥ 1.312

Model 1: All covariates were not adjusted

Model 2: Adjusted for age and gender

Model 3: Adjusted for age, gender, hip circumference, systolic blood pressure, diastolic blood pressure, fasting blood glucose, 2-h postprandial blood glucose, glycosylated hemoglobin, high-density lipids protein, triglyceride, serum creatinine, serum uric acid, urine albumin-creatinine ratio, Mini-Mental State Examination, smoking history, and drinking history

*HR* Hazard ratio, *CI* Confidence interval

^*^P < 0.05

**Fig. 4:**
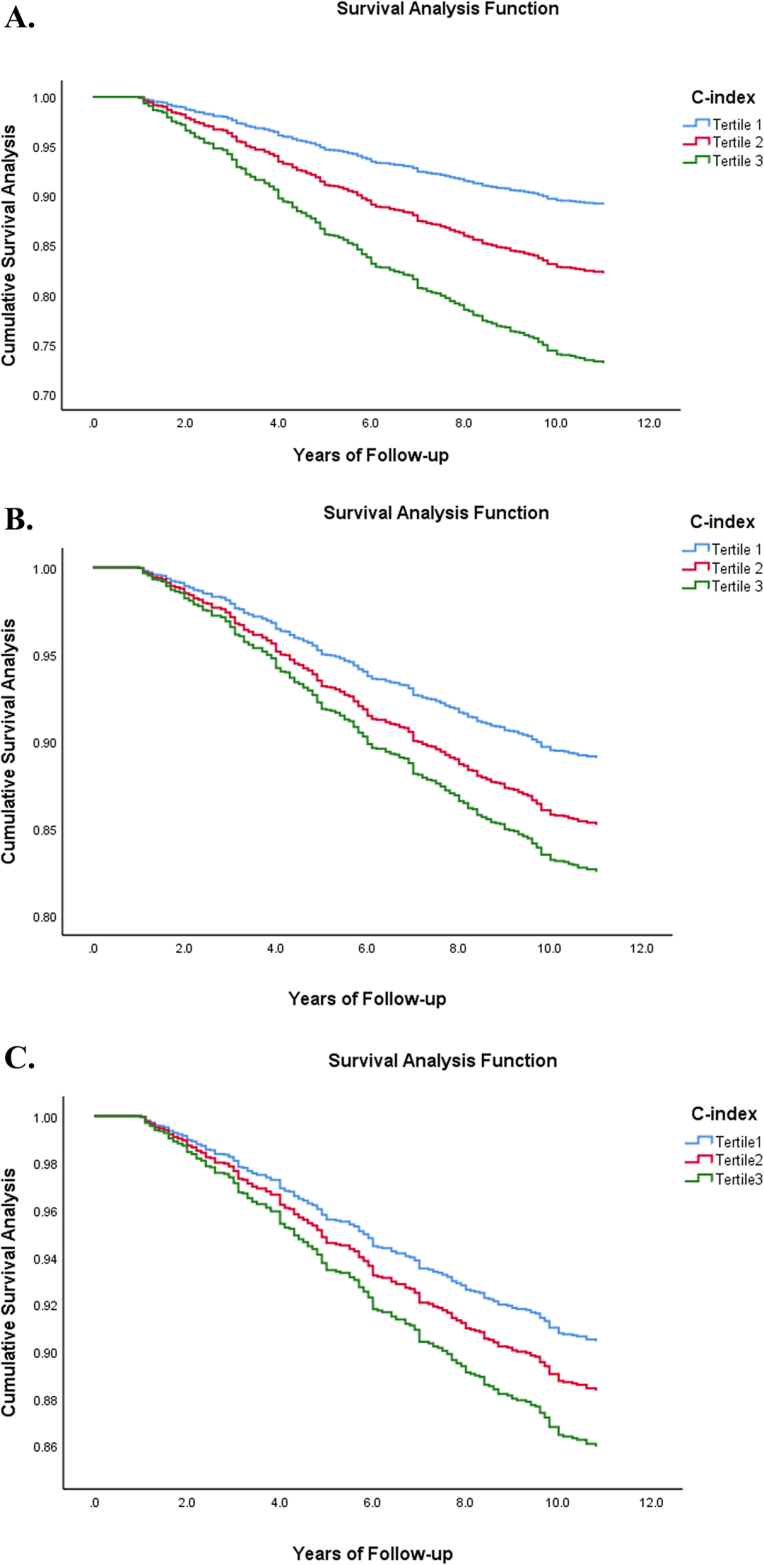
Cox survival analysis of C-index and all-cause mortality. Tertile 1: C-index < 1.249; Tertile 2: 1.249 ≤ C-index < 1.312; Tertile 3: C-index ≥ 1.312. **A** Model 1: All covariates were not adjusted. **B** Model 2: Adjusted for age and gender. **C** Model 3: Adjusted for age, gender, hip circumference, systolic blood pressure, diastolic blood pressure, fasting blood glucose, 2-h postprandial blood glucose, glycosylated hemoglobin, high-density lipids protein, triglyceride, serum creatinine, serum uric acid, urine albumin-creatinine ratio, Mini-Mental State Examination, smoking history, and drinking history

As shown in Fig. 4, in the Cox proportional hazards model, survival analysis curves were made for the three models of C-index. It can also be seen from the survival analysis curves of the three models that there are significant differences in the survival rates of different C-index groups. Groups with higher C-index values had a higher risk of death.

### Hierarchical analysis

**Fig. 5:**
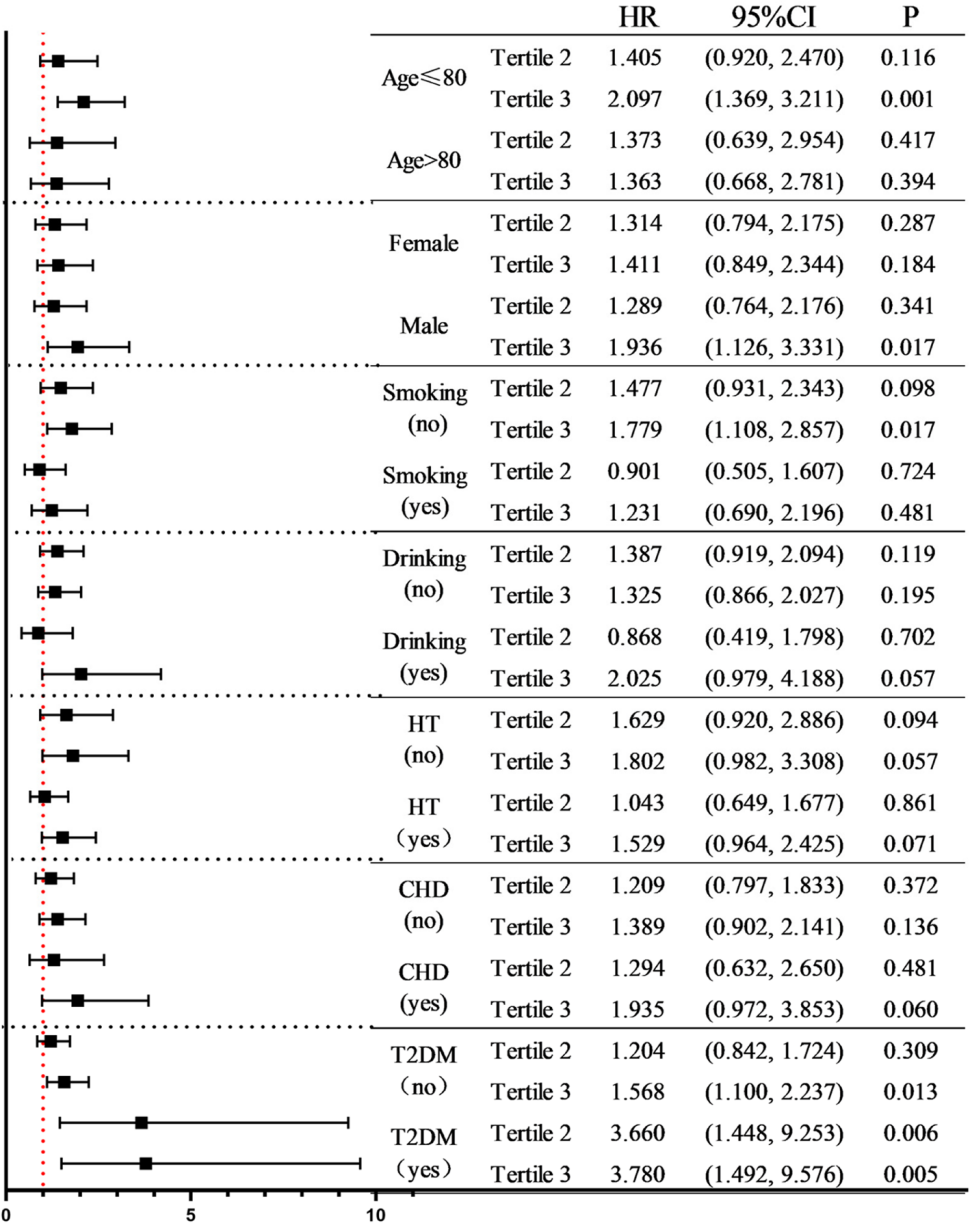
The relationship between C-index and all-cause mortality under different stratifications. Tertile 1: C-index < 1.249; Tertile 2: 1.249 ≤ C-index < 1.312; Tertile 3: C-index ≥ 1.312. CHD: Coronary Heart Disease; HT: Hypertension; T2DM: Type 2 diabetes mellitus. Stratified analysis by age, gender, smoking history, drinking history, history of HT, history of CHD, and history of T2DM. Adjusted for age, gender, hip circumference, systolic blood pressure, diastolic blood pressure, fasting blood glucose, 2-h postprandial blood glucose, glycosylated hemoglobin, high-density lipids protein, triglyceride, serum creatinine, serum uric acid, urine albumin-creatinine ratio, Mini-Mental State Examination, smoking history, and drinking history

A stratified analysis of covariates with a greater effect on all-cause mortality in the COX risk model was performed, stratified by the following variables: age, gender, smoking history, drinking history, history of HT, history of CHD, and history of T2DM. After controlling for all covariates except stratification variables, the association between C-index and risk of all-cause mortality remained in subgroup analyses. Figure 5 shows the forest plot of the stratified analysis, and the results show that the third tertile of the C-index and all-cause mortality were all statistically significant in the population aged ≤ 80 years, male, and with no smoking history. In addition, we stratified separately for the presence or absence of a history of HT, CHD, and T2DM. In the population with T2DM, with Tertile 1 as the reference, the risk of all-cause mortality in the second and third tertiles was 3.660 and 3.780 times that of Tertile 1, and the difference was statistically significant. Among people without diabetes, the risk of all-cause mortality differed only in the third tertile, using Tertile 1 as a reference. In the population with a history of HT and CHD, the results of the stratified analysis were not statistically significant.

## Discussion

The purpose of this study was to determine the ability of the C-index to predict all-cause mortality in Chinese older people. Using an non-cancer older population in a Beijing community, we conducted nearly 10 years of follow-up. We found that C-index was associated with and predicted the risk of all-cause mortality in this population. The Cox regression analysis showed that C-index is an independent risk factor for all-cause death in older people in community. Especially among people with diabetes, older people with a high C-index had a higher risk of all-cause death.

Obesity is critical to the health of the aging population. Excessive obesity, especially abdominal obesity, has long-term negative effects on cardiovascular outcomes and mortality [27]. However, the relationship between obesity and mortality in older people is controversial [28]. On the other hand, in some recent reports defined as the "obesity paradox", older people who are overweight or mildly obese may be more favorable than underweight [29, 30]. Therefore, it is particularly important to further clarify the relationship between obesity and the long-term survival of older people. In this study of older people in the Chinese community, the C-index was more predictive of all-cause mortality than other obesity indicators, with an AUC of 0.633. Second, the AUC of C-index was greater than that of women in male community elders. By Cox proportional risk model analysis, we analyzed the ability of C-index to predict all-cause mortality among older people in the community. Our results suggest that C-index can be used as an independent risk factor for all-cause mortality. Previous studies on C-index mainly focused on the risk of death from coronary heart disease (CHD) [31], and there was no research on the relationship between C-index and all-cause death. In 2014, Tonding et al. found in a study of obesity markers and coronary heart disease risk in diabetic patients that C-index was the body obesity marker most associated with a higher risk of fatal CHD in these diabetic patients [32]. However, other studies have had mixed results [33]. The Framingham Heart Study by Kim et al. showed that C-index was not associated with increased CHD morbidity or mortality [34]. BMI is a better predictor of CHD morbidity and mortality than C-index. In contrast to our study, the Framingham cohort included non-institutionalised white men and women aged between 30 and 62 years. During 24 years of follow-up, 248 men and 150 women died from coronary heart disease-related causes. Factors such as age, race and gender may all have had a differential impact on the results. To further demonstrate the association between C-index and all-cause death, we performed a stratified analysis, stratified by age, gender, smoking history, drinking history, history of HT, history of CHD, and history of T2DM. After controlling for all covariates except stratified variables, the association between C-index and risk of all-cause mortality persisted in the subgroup analysis. The association between the third tertile of the c index and all-cause mortality was statistically significant in subjects aged ≤ 80 years, or in subjects with no history of smoking, or in male subjects. In addition, we stratified separately for the presence or absence of diabetes. Furthermore, we stratified the presence or absence of diabetes separately. Among diabetic patients, the second and third tertiles had a statistically significant 3.660 and 3.780 times higher risk of all-cause mortality than Tertile 1, respectively. In people without diabetes, using Tertile 1 as a reference, the difference in risk of all-cause mortality was only statistically significant in the third tertile. It can be seen that in the diabetic population, patients with abdominal obesity have a high risk of death.

Aging not only promotes body fat gain but also changes its distribution. This fat distribution is characterized by a decrease in subcutaneous fat in the gluteal femoral region, which reduces the ability of subcutaneous adipocytes to store body fat. Thus, circulating free fatty acids from ectopic fat deposits in older people are increased. Increased visceral, intrahepatic, and intramuscular fat can lead to insulin resistance and metabolic changes [35]. Abdominal fat distribution is associated with increased insulin resistance and risk of T2DM and cardiovascular disease [36]. Fat accumulation is associated with pro-inflammatory cytokines, oxidative stress, and insulin resistance, and also contributes to muscle fiber atrophy and mitochondrial dysfunction, potentially contributing to the development and progression of sarcopenia [23]. Insulin resistance and hyperinsulinemia due to abdominal obesity were also associated with increased aortic stiffness [37, 38]. These studies have proved that abdominal obesity is significantly harmful to older people, which also promotes the occurrence of all-cause mortality in older people. However, the mechanism of various diseases in older people with abdominal obesity is still not well studied, and more basic research on abdominal obesity should be paid attention to.

### Limitations and strengths of the study

C-index is a parameter that takes into account WC, weight, and height. In European and American populations, it is considered to be a health indicator comparable to the waist-to-hip ratio (WHR) [39]. However, there are few studies on the application of C-index in China. The C-index has a theoretical range that includes built-in adjustments for height and weight, allows direct comparison of abdominal adiposity between individuals and even between populations, and does not require hip circumference to assess fat distribution. Another strength of our study is that we have a mean follow-up of 9.87 years, and the results obtained with longer follow-up are more reliable. In addition, our research population is older people in community. In today's aging world, deepening the research on older people in community is of great significance to the health management and disease prevention of older people. Our study also has some limitations. First, there is a lack of accurate measurements of body composition determined by dual energy X-ray absorptiometry (DXA) or magnetic resonance imaging, which are capable of distinguishing abdominal visceral fat from subcutaneous fat. Second, 263 people were lost to follow-up during our study. Although the lost population only accounted for 12.2% of the study population, it may also have a certain impact on the results.

## Conclusions

In conclusion, our study shows that C-index is an independent risk factor for all-cause mortality among non-cancer older people in the community, especially in the diabetic population, patients with abdominal obesity had a high risk of all-cause death.

## Data Availability

The research data used to support the findings of this study are available from the corresponding authors upon request.

## Abbreviations

AO: Abdominal obesity
T2DM: Type 2 diabetes mellitus
C-index: Conicity-index
ROC: Receiver operating characteristic
CT: Computed tomography
BMI: Body mass index
WC: Waist circumference
AVI: Abdominal volume index
BRI: Body roundness index
LAP: Lipid accumulation products
WHtR: Waist-height ratio
FBG: Fasting blood glucose
2hPG: 2-Hour postprandial blood glucose
HDL: High-density lipoprotein
LDL: Low-density lipoprotein
NIHSS: National Institutes of Health Stroke Scale
MMSE: Mini-Mental State Examination
ADL: Activity of Daily Living
HC: Hip circumference
ANOVA: A one-way analysis of variance
AUC: Area under the curve
HR: Hazard ratios
CI: Confidence intervals
CHD: Coronary heart disease
WHR: Waist-to-hip ratio
DXA: Dual energy X-ray absorptiometry
